# AST/ALT ratio, APRI, and FIB-4 compared to FibroScan for the assessment of liver fibrosis in patients with chronic hepatitis B in Bandar Abbas, Hormozgan, Iran

**DOI:** 10.1186/s12876-023-02780-w

**Published:** 2023-05-11

**Authors:** Seyed Hamid Moosavy, Ebrahim Eftekhar, Parivash Davoodian, Abdolazim Nejatizadeh, Mohammad Shadman, Shahram Zare, Mirza Ali Nazarnezhad

**Affiliations:** 1 Department of Internal Medicine, Infectious and Tropical Disease Research Center, Hormozgan Health Institute, Hormozgan University of Medical Sciences, Bandar Abbas, Hormozgan, Iran; 2 Molecular Medicine Research Center, Hormozgan Health Institute, Hormozgan University of Medical Sciences, Bandar Abbas, Hormozgan, Iran; 3 Infectious and Tropical Disease Research Center, Hormozgan Health Institute, Hormozgan University of Medical Sciences, Bandar Abbas, Hormozgan, Iran; 4 Hormozgan University of Medical Sciences, Bandar Abbas, Hormozgan, Iran

**Keywords:** APRI, AST/ALT ratio, Chronic hepatitis B, FIB-4, Fibroscan

## Abstract

**Background:**

Chronic hepatitis B (CHB) is a significant risk factor for liver-related disorders. Hepatic fibrosis staging by liver biopsy in these patients can lead to complications. This study aimed to compare aspartate aminotransferase/alanine aminotransferase (AST/ALT) ratio, AST to platelet ratio index (APRI), and fibrosis-4 (FIB-4) with FibroScan results for the evaluation of hepatic fibrosis in CHB patients.

**Methods:**

This cross-sectional study included patients with CHB referred to the outpatient clinics of Bandar Abbas, Hormozgan, Iran, in 2021. The age and sex of the participants were noted. FibroScan evaluation was done for all subjects. Moreover, AST, ALT, and platelet counts were measured in their blood samples within one month of the FibroScan evaluation.

**Results:**

Of the 267 CHB patients evaluated in the present study (mean age: 45.45 ± 18.16 years), 173 (64.8%) were male. According to FibroScan results, 65 CHB patients (24.3%) had F1, 53 (19.9%) F2, 38 (14.2%) F3, and 20 (7.5%) F4 liver fibrosis. There was a significant correlation between FibroScan results and the three indices of AST/ALT ratio, APRI, and FIB-4 (P < 0.001), with the strongest correlation between FibroScan results and APRI (r = 0.682). With an area under the receiver operating characteristic (AUROC) curve of 0.852 (95% confidence interval [CI] 0.807; 0.897, P < 0.001), APRI ≥ 0.527 had the best diagnostic accuracy (77.15%) for the detection of any grade of liver fibrosis. Although the AUROC curve of APRI and FIB-4 was similar (0.864) for distinguishing between F3/F4 and F0-F2 of liver fibrosis, FIB-4 had the best diagnostic accuracy (82.02%).

**Conclusions:**

APRI can rule out 95.4% of F3/F4 of liver fibrosis and rule in any grade of liver fibrosis in CHB patients by 90.78%. Therefore, APRI appears to be the best substitute for FibroScan in the assessment of liver fibrosis in patients with CHB.

## Introduction

Affecting 292 million individuals worldwide, chronic hepatitis B (CHB) is a significant risk factor for liver-related disorders, such as hepatocellular carcinoma, cirrhosis, and liver failure [1, 2]. According to an updated systematic review and meta-analysis, the prevalence of hepatitis B virus (HBV) infection in Iran is 2.2% [3]. Additionally, based on a recent study conducted in the Iranian province of Hormozgan, 2.5% of the sample population tested positive for HBV surface antigen, whereas, 11.6% were positive for anti-HBV core antibodies [4].

Hepatic fibrosis staging is crucial for treating CHB patients in clinical practice because patients with severe liver fibrosis have a greater risk of complications. While liver biopsy is the gold standard for diagnosing liver fibrosis, it is not often approved by either patients or physicians due to its invasive nature, sampling inaccuracies, and other limitations [5]. Therefore, alternative non-invasive techniques have received a lot of attention, including transient elastography [6]. Nevertheless, because of the expensive cost of equipment, particularly for regions with limited resources, transient elastography (FibroScan) has not yet been generally accessible. Formulas based on blood tests from standard hematological and biochemical testing are less costly and more readily available, and they appear to be the best substitutes for liver biopsy. Among these, the aspartate aminotransferase/alanine aminotransferase (AST/ALT) ratio, AST to platelet ratio index (APRI), and fibrosis-4 (FIB-4), approved by expert consensus and different clinical recommendations, have been widely utilized to stage hepatic fibrosis in CHB [7].

The diagnostic values of AST/ALT ratio, APRI, and FIB-4 for different stages of liver fibrosis have been the subject of several investigations [8–14]. However, most of these studies have determined the diagnostic thresholds of the above-mentioned indices based on sensitivity and specificity, which can only serve as references when choosing a diagnostic test. The number of patients properly ruled in or out by these indices is what matters most in clinical practice, which lies under the definition of positive predictive value (PPV) and negative predictive value (NPV), respectively [5, 7]. Thus, this study aimed to compare AST/ALT ratio, APRI, and FIB-4 to FibroScan results for the evaluation of hepatic fibrosis in CHB patients, as well as to determine the optimal diagnostic threshold and diagnostic values for each index.

## Methods

### Participants

This cross-sectional study included patients with CHB referred to outpatient clinics of Bandar Abbas, Iran, in 2021. Chronic hepatitis B was diagnosed based on the laboratory test results for HBV markers by an experienced gastroenterologist. Patients were only included in this research if they provided written informed consent. This research conforms with the principles of the Declaration of Helsinki and was granted ethical clearance by the Ethics Committee of Hormozgan University of Medical Sciences (IR.HUMS.REC.139.344). Patients with incomplete demographic or clinical information, and other chronic liver diseases, including non-alcoholic fatty liver disease, alcoholic liver disease, autoimmune hepatitis, hepatitis C, and hepatitis D, were excluded. Moreover, patients using hepatotoxic medications such as methotrexate, those with congestive heart failure, hepatic congestion, decompensated cirrhosis based on clinical or ultrasound evidence, increased body mass index interfering with FibroScan evaluation, and those taking hormonal or herbal medications were excluded. Patients were recruited through convenience sampling.

### Study design

The required data were recorded using a checklist. Age and sex were the demographic characteristics of the patients that were noted. Then, all patients underwent FibroScan using the same FibroTouch 502 device (Echosens, France) by a single gastroenterologist. All FibroScans were carried out in accordance with the manufacturer’s instructions. Results from FibroScan were categorized as follows in light of prior research and manufacturer recommendations [15]:

- F0: 1–6 kPa.
- F1: 6.1-7 kPa.
- F2: 7.1-9 kPa.
- F3: 9.1–10.3 kPa.
- F4: ≥10.4 kPa.

Within a month of the FibroScan examination, the following measures were made in random blood samples collected from all patients:

- Serum ALT with 45.25 U/L as the upper limit of normal in men and 30.47 in women.
- Serum AST with 15–37 U/L as the normal range.
- Platelet count with 150,000-400,000 /µl as the normal range.

Blood samples were collected from all patients and their sera were used for AST and ALT assays. The enzymatic activity was determined using standard kits (Pars Azmoon Co., Tehran, Iran) and the chemistry autoanalyzer BT1500 (Biiotechnical Instruments, Rome, Italy). The platelet count was measured using Mindray BC 3000 automatic hematology analyzer (Mindary Corp., China) according to the manufacturer’s protocol. AST/ALT ratio was calculated for each patient. APRI and FIB-4 were also calculated based on the following formulas:

$$\text{A}\text{P}\text{R}\text{I}=\frac{\frac{\text{A}\text{S}\text{T} \text{l}\text{e}\text{v}\text{e}\text{l}}{\text{A}\text{S}\text{T} \text{U}\text{L}\text{N} \left(\text{u}\text{p}\text{p}\text{e}\text{r} \text{l}\text{i}\text{m}\text{i}\text{t} \text{o}\text{f} \text{n}\text{o}\text{r}\text{m}\text{a}\text{l}\right)}}{\text{P}\text{l}\text{a}\text{t}\text{e}\text{l}\text{e}\text{t} \text{c}\text{o}\text{u}\text{n}\text{t} \left({10}^{9}/\text{L}\right)}\times 100$$

$$\text{F}\text{I}\text{B}-4=\frac{\text{A}\text{g}\text{e} \left(\text{y}\text{e}\text{a}\text{r}\text{s}\right)\times \text{A}\text{S}\text{T} (\text{U}/\text{L})}{\text{P}\text{l}\text{a}\text{t}\text{e}\text{l}\text{e}\text{t} \text{c}\text{o}\text{u}\text{n}\text{t} \left({10}^{9}/\text{L}\right)\times \sqrt{\text{A}\text{L}\text{T} (\text{U}/\text{L})}}$$

### Data analysis

The Statistical Package for the Social Sciences (SPSS) software (version 25.0, Armonk, NY: IBM Corp.) was used for data analysis. Mean, standard deviation, frequency, and percentages were used to describe the variables. Distribution normality of continuous variables were determined using the Kolmogorov-Smirnov normality test. Accordingly, Spearman’s correlation was used to determine their correlations. Based on the central limit theorem and the sample size of more than 30 in each group [16], the independent t-test was used for the comparison of indices between males and females. Because the variances were not homogeneous, we used the Welch robust test of equality of means for comparison of AST/ALT ratio, APRI, and FIB-4 by different stages of liver fibrosis. Accordingly, the Tamhane test was used for pair-wise post-hoc analysis.

To distinguish between F1-F4 and F0 of liver fibrosis, as well as F3/F4 and F0-F2, the diagnostic values of AST/ALT ratio, APRI, and FIB-4 were determined using receiver operating characteristic (ROC) curves. The area under the ROC (AUROC) curves were calculated for each non-invasive index. The AUROCs were compared using the roccomp command in Stata (version 14.2). The optimal cut-offs of all three indices were also determined for this purpose, using the ROC curves and the maximum Youden’s index. Sensitivity, specificity, PPV, NPV, and diagnostic accuracy (DA) were estimated for these cut-offs as well. P-values < 0.05 were regarded as statistically significant.

## Results

Of the 267 CHB patients evaluated in the present study, 173 (64.8%) were male and 94 (35.2%) were female. The mean age of the participants was 45.45 ± 18.16 years. According to FibroScan results, 65 CHB patients (24.3%) had F1, 53 (19.9%) F2, 38 (14.2%) F3, and 20 (7.5%) F4 liver fibrosis (Table 1).

**Table 1:** General characteristics of the study participants

Variables	Values
Age (years), mean (SD)	45.45 (18.16)
Sex, N (%)	
Male	173 (64.8)
Female	94 (35.2)
AST (U/L), mean (SD)	39.46 (16.26)
ALT (U/L), mean (SD)	41.13 (13.13)
Platelet count (/µl), mean (SD)	171868.91 (59279.65)
APRI, mean (SD)	0.744 (0.533)
FIB-4, mean (SD)	2.03 (1.76)
AST/ALT ratio, mean (SD)	0.97 (0.30)
FibroScan results, N (%)	
F0	91 (34.1)
F1	65 (24.3)
F2	53 (19.9)
F3	38 (14.2)
F4	20 (7.5)

Abbreviations: ALT, alanine aminotransferase; APRI, AST to platelet ratio index; AST, aspartate aminotransferase; FIB-4, fibrosis-4; N, number; SD, standard deviation

There was a significant correlation between FibroScan results and the three indices of AST/ALT ratio, APRI, and FIB-4 (P < 0.001); with the strongest correlation observed between FibroScan results and APRI (r = 0.682) (Table 2). Comparison of FibroScan results and different indices between men and women showed no significant differences (Table 3).

**Table 2:** Correlation of different indices with FibroScan results

First variable	Second variable	Correlation coefficient	P-value*
FibroScan results	AST/ALT ratio	0.217	< 0.001
	APRI	0.682	< 0.001
	FIB-4	0.626	< 0.001

Abbreviations: ALT, alanine aminotransferase; APRI, AST to platelet ratio index; AST, aspartate aminotransferase. FIB-4, fibrosis-4

*Analyzed by Spearman’s correlation

**Table 3:** Comparison of different indices by sex

Indices	Male (n = 173)	Female (n = 94)	P-value*
FibroScan results, N (%)			
F0	56 (32.4)	35 (37.2)	0.590†
F1	42 (24.3)	23 (24.5)	
F2	36 (20.8)	17 (18.1)	
F3	28 (16.2)	10 (10.6)	
F4	11 (6.4)	9 (9.6)	
AST/ALT ratio, mean (SD)	0.96 (0.29)	0.99 (0.31)	0.512
APRI, mean (SD)	0.752 (0.495)	0.730 (0.598)	0.744
FIB-4, mean (SD)	2.07 (1.73)	1.95 (1.81)	0.582

Abbreviations: ALT, alanine aminotransferase; APRI, AST to platelet ratio index; AST, aspartate aminotransferase; FIB-4, fibrosis-4; N, number; SD, standard deviation

*Analyzed by the independent t-test

†Analyzed by Chi-squared test

There was a significant difference in AST/ALT ratio, APRI, and FIB-4 between different stages of liver fibrosis by FibroScan (P < 0.001). However, post-hoc analysis showed that the difference in APRI between F3 and F4 was not statistically significant (P = 0.051). Moreover, the difference in FIB-4 between F0 and F1 (P = 0.531) as well as F3 and F4 (P = 0.085) was not statistically significant. Also, for AST/ALT ratio, F0-F1 (P = 0.842), F0-F2 (P = 1.000), F1-F2 (P = 0.767), F2-F3 (P = 0.078), and F3-F4 (P = 0.947) were not significantly different (Table 4).

**Table 4:** APRI, FIB-4, and AST/ALT ratio by FibroScan results

FibroScan results	APRI	FIB-4	AST/ALT ratio
F0	0.408 (0.189)	1.16 (0.91)	0.92 (0.16)
F1	0.610 (0.282)	1.41 (0.83)	0.87 (0.23)
F2	0.798 (0.321)	2.18 (1.05)	0.94 (0.23)
F3	1.163 (0.510)	3.23 (1.69)	1.14 (0.42)
F4	1.774 (0.820)	5.31 (3.04)	1.29 (0.47)
P-value*	< 0.001	< 0.001	< 0.001

Abbreviations: ALT, alanine aminotransferase; APRI, AST to platelet ratio index; AST, aspartate aminotransferase; FIB-4, fibrosis-4; N, number; SD, standard deviation

All values are expressed as mean (SD).

*Analyzed by the Welch robust test of equality of means

**Fig. 1:**
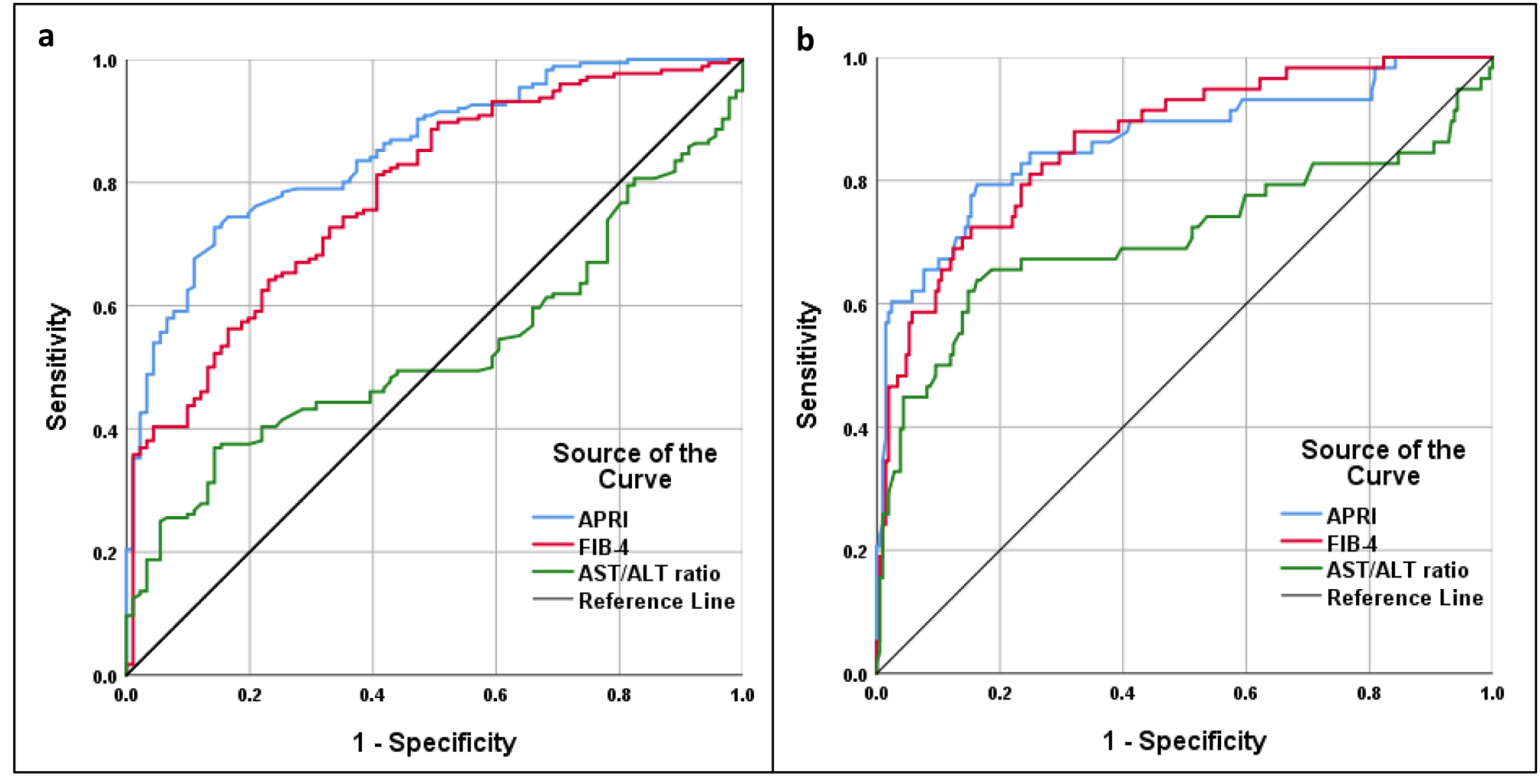
**a)** Receiver operating characteristic curves of AST/ALT ratio, APRI, and FIB-4 for the detection of F1-F4 of liver fibrosis from F0, and **b)** F3/F4 from F0-F2 of liver fibrosis

Figure 1 shows the ROC curves of AST/ALT ratio, APRI, and FIB-4 to distinguish between F1-F4 and F0 of liver fibrosis, as well as F3/F4 and F0-F2. For the detection of any grade of liver fibrosis (F1-F4), the highest sensitivity and specificity belonged to FIB-4 (81.25%) and AST/ALT ratio/FIB-4 (85.71%), respectively. Meanwhile, APRI (90.78%) and FIB-4 (62.07%) had the highest PPV and NPV for this purpose. Overall, with an AUROC curve of 0.852 (95% confidence interval [CI] 0.807; 0.897, P < 0.001), APRI ≥ 0.527 had the best diagnostic accuracy (77.15%) (Table 4). There was a significant difference regarding AUROCs of AST/ALT ratio, APRI, and FIB-4 (P < 0.001).

As for the detection of F3/F4 from F0-F2, APRI (89.66%) and FIB-4 (84.69%) had the highest sensitivity and specificity, respectively. For PPV and NPV it was the other way around; FIB-4 (57.76%) and APRI (95.35%) showed the highest PPV and NPV. Although the AUROC curve of APRI and FIB-4 was similar (0.864), FIB-4 (82.02%) had the best diagnostic accuracy (Table 5). The AUROC of APRI and FIB-4 were both significantly higher than that of AST/ALT ratio (P < 0.001), but there was no significant difference between AUROCs of APRI and FIB-4 (P = 0.978).

**Table 5:** Diagnostic performance of the indices for the differentiation of F3 and F4 from lower stages

	Indices	AUC (95% CI)	P-value	Cut-off	Sensitivity (%)	Specificity (%)	PPV (%)	NPV (%)	DA (%)
F0 and F1-F4	AST/ALT ratio	0.529 (0.461; 0.598)	< 0.001	1.07	36.93	85.71	83.33	41.27	53.56
	APRI	0.852 (0.807; 0.897)	< 0.001	0.527	72.73	85.71	90.78	61.90	77.15
	FIB-4	0.783 (0.727; 0.840)	0.431	1.11	81.25	59.34	79.44	62.07	73.78
F0-F2 and F3/F4	AST/ALT ratio	0.710 (0.616; 0.804)	< 0.001	1.08	63.79	83.73	52.11	89.29	79.40
	APRI	0.864 (0.802; 0.926)	< 0.001	0.536	89.66	58.85	37.68	95.35	65.54
	FIB-4	0.864 (0.811; 0.918)	< 0.001	2.15	72.41	84.69	57.76	91.71	82.02

Abbreviations: ALT, alanine aminotransferase; APRI, AST to platelet ratio index; AST, aspartate aminotransferase; AUC, area under the curve; CI, confidence interval; DA, diagnostic accuracy; FIB-4, fibrosis-4; NPV, negative predictive value; PPV, positive predictive value

## Discussion

The current study’s findings demonstrated that APRI can rule in any grade of liver fibrosis (with a PPV of 90.8%) and rule out F3/F4 of liver fibrosis (with an NPV of 95.4%) in CHB patients. Moreover, this index showed the highest AUROC compared to AST/ALT ratio and FIB-4 for the detection of any grade of liver fibrosis and superior AUROC than AST/ALT ratio but similar AUROC to FIB-4 for distinguishing between F3/F4 and F0-F2 of liver fibrosis in these patients.

Chronic hepatitis B is still widespread worldwide [1]. The treatment, monitoring, and prognosis of CHB patients depend on an accurate diagnosis of hepatic fibrosis [5, 7]. Non-invasive diagnostic techniques have attracted a lot of attention in recent years as means of determining the stage of hepatic fibrosis; nevertheless, a large number of CHB patients without fibrosis were mistakenly diagnosed as having significant fibrosis by the most commonly used indices in clinical practice, proving that these indices were ineffective in predicting CHB-related liver fibrosis [17]. A false-positive result may lead to early or unnecessary use of antiviral medications and the accompanying risk of drug toxicity and possible drug resistance [18]. On the other hand, given the potential risks of liver biopsy and the cost of FibroScan in areas with limited resources, non-invasive hepatic fibrosis assessment is necessary [19].

In agreement with our findings, nearly two-thirds of Chinese patients with CHB had a decreased requirement for FibroScan based on the APRI’s correct assessment of liver fibrosis [11]. Yue et al. reported that an APRI cut-off of 0.8 detected bridging fibrosis (F ≥ 3) [11]. Additionally, Sha et al. stated that APRI was the most accurate non-invasive index to predict F2/F3 liver fibrosis compared to FIB-4, AST/ALT ratio, and AST/ALT/platelet ratio [12]. The World Health Organization (WHO) has also advised the APRI index for assessing liver fibrosis in CHB with a threshold of 0.5–1.5 for significant fibrosis [18]. The APRI cut-off of the present study for F3/F4 liver fibrosis was 0.536 which is within the above-mentioned range; nonetheless, screening for FibroScan using these WHO cut-offs missed a large number of individuals with significant fibrosis. Contrarily, our cut-off yielded an approximately 90% sensitivity in this respect, while APRI values lower than 0.536 were able to rule out F3/F4 liver fibrosis by an NPV of 95.4%.

In another study, the AUROC of FIB-4 at a cut-off of 1.571 was 0.82 for severe liver fibrosis [13], while ours at a cut-off of 2.15 was 0.84. Further, with similar AUROC of FIB-4 and APRI in the current study for differentiating F3/F4 from F0-F2 liver fibrosis, FIB-4 yielded a higher specificity. Studies on non-alcoholic fatty liver disease and chronic hepatitis C have also shown acceptable diagnostic values of APRI for the detection of significant liver fibrosis [20, 21]. Similarly, Alhankawi et al. showed that APRI was comparable with FIB-4 but superior to AST/ALT ratio for predicting significant liver fibrosis in hepatitis C patients. However, these indices were more beneficial in ruling out than ruling in significant fibrosis in these patients [22].

It is noteworthy that FibroScan results may be less accurate in patients with obesity or ascites, as the presence of excess tissue or fluid may interfere with the accuracy of the test [23]. In addition, the accuracy of FibroScan results may depend on the operator’s experience and technique, as proper positioning of the device on the skin is critical for accurate results [24]. Moreover, FibroScan results may also vary between different devices leading to variability in diagnosis and treatment decisions [25]. However, we tried to address these issues by using the same device for all our patients, and all evaluations were done by the same operator to avoid inter-observer variability. Furthermore, inflammation can cause false-positive FibroScan results, leading to an overestimation of the liver fibrosis stage [26].

The use of FibroScan rather than liver biopsy placed certain restrictions on this study. Liver biopsy is considered the gold standard for the diagnosis of liver fibrosis, but it also has several limitations, including invasiveness, the risk of complications such as bleeding, pain, infection, and injury to other organs, sampling error which means that the small tissue obtained may not accurately represent the overall condition of the liver, and absolute or relative contraindications in patients with bleeding disorders, ascites, or other medical conditions [27]. However, research has shown strong agreement between liver biopsy and FibroScan in CHB patients [28]. FibroScan’s volume measurements of the liver’s mass are about a hundred times more accurate than those of biopsy specimens, making FibroScan results more indicative of the total hepatic parenchyma [29]. Furthermore, the relatively small sample size can limit the generalizability of our findings. In addition, hepatitis B activity may have influenced the results which were not accounted for in the current study. Also, there is a more recent liver fibrosis score, FIB-5, that incorporates albumin levels that could not be calculated for our patients because albumin levels were not evaluated.

## Conclusions

By comparing APRI with FIB-4 and AST/ALT ratio, we discovered that APRI had the strongest correlation with the findings of FibroScan and was the most effective marker for ruling out advanced liver fibrosis. Moreover, it was the best index to rule in any grade of liver fibrosis in CHB patients. Thus, in order to evaluate liver fibrosis in CHB patients, APRI seems to be the best FibroScan alternative, aiding in the choice of further potentially invasive tests, refer patients to higher levels of care, and recommend lifestyle changes. Future studies should take body mass index, hepatitis B activity, and FIB-5 scores into consideration.

## Data Availability

The datasets used and/or analyzed during the current study are available from the corresponding author on reasonable request.

## List of abbreviations

ALT: Alanine aminotransferase
APRI: AST to platelet ratio index
AST: Aspartate aminotransferase
AUROC: Area under the receiver operating characteristic curve
CHB: Chronic hepatitis B
DA: Diagnostic accuracy
FIB-4: Fibrosis-4
HBV: Hepatitis B virus
NPV: Negative predictive value
PPV: Positive predictive value
ROC: Receiver operating characteristic
SPSS: Statistical Package for the Social Sciences
WHO: World Health Organization
